# Managing type 2 diabetes: targeting a microbial enzyme as a novel treatment option

**DOI:** 10.1038/s41392-023-01694-z

**Published:** 2023-12-06

**Authors:** Anikó Keller-Pintér, Tamás Korcsmáros, Tibor Vellai

**Affiliations:** 1 Department of Biochemistry, Albert Szent-Györgyi Medical School, University of Szeged, Szeged, 6720 Hungary; 2 Department of Metabolism, Digestion and Reproduction, Imperial College London, London, W12 0NN UK; 3 Quadram Institute Bioscience, Norwich Research Park, Norwich, NR4 7UQ UK; 4 Department of Genetics, Eötvös Loránd University (ELTE), Budapest, 1117 Hungary; 5 Hungarian Research Network HUN-REN-ELTE Genetics Research Group, Budapest, 1117 Hungary

**Keywords:** Molecular medicine, Structural biology

In a recent *Science* article, Wang and colleagues showed that the dipeptidyl peptidase 4 (DPP4) enzyme produced by the gut microbiota can interfere with the effect of host glucagon-like peptide-1 (GLP-1) involved in stimulating insulin secretion, and the specific inhibitor of microbial DPP4 (mDPP4), combined with clinical inhibitors of host DPP4 isoenzymes, further promotes blood glucose homeostasis.^1^ These results point out why certain patients respond inadequately to an antidiabetic medication.

Type 2 diabetes (T2D), also known as adult-onset diabetes, is a common metabolic disorder characterized by the inability of cells to respond properly to insulin hormone mediating cellular glucose uptake and/or insufficient insulin production by pancreatic beta cells. As a consequence, the affected patients exhibit elevated levels of blood glucose (hyperglycemia), so their cells degrade other substances besides glucose (sugar), like lipids, to gain energy. The pathology affects millions worldwide (for example, around 40% of young adults in the US are influenced by insulin resistance, an early stage of T2D, and its prevalence increases with age^2^), and, currently, there is no cure for T2D. Beyond lifestyle interventions, diabetes medications or insulin therapy are used to help manage the disease. Among the antidiabetic drugs, metformin is frequently applied, but specific DPP4 inhibitors including alogliptin, sitagliptin, saxagliptin and linagliptin^3^ can also be utilized. A serious limitation of using these drugs is the inability of certain individuals to respond to the treatment.

**Fig. 1:**
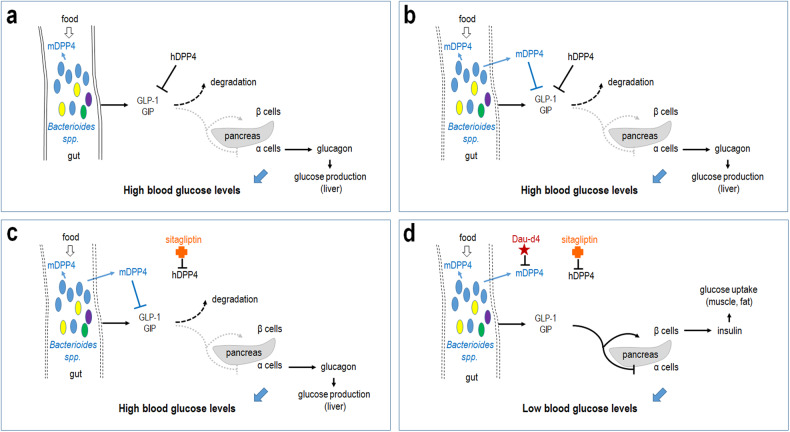
Mechanistic model for how co-administration of specific microbial and host DPP4 inhibitors effectively lowers blood glucose levels. **a** Under normal conditions, microbial DPP4 (mDPP4—blue) produced by the gut microbiota cannot penetrate through the intestinal wall. Its host isozyme, hDPP4 (black), inhibits GLP-1 and GIP hormones called incretins, which normally are created by intestinal cells in response to nutrition, and stimulate insulin secretion and inhibit glucagon release from the pancreatic β and α cell, respectively (dotted, light gray arrows and bars), thereby leading to decreased levels of blood glucose (insulin lowers, whereas glucagon elevates, blood glucose levels), indicated by the thick, blue arrow. **b** Under certain conditions, mDPP4 can penetrate through the intestinal wall and functions redundantly with hDPP4. **c** When hDPP4 is specifically inhibited by sitagliptin (orange cross), mDPP4 can still block incretins. Thus, blood glucose levels remain high. **d** Simultaneous hampering both host and microbial DPP4 isozymes (hDPP4 and mDPP4) by co-administration of sitagliptin and Dau-d4 (a specific inhibitor for mDPP4—purple star) effectively activates GLP-1 and GIP, thereby enhancing the first-phase insulin response (blood glucose levels lower significantly). Dashed lines represent permeable intestinal wall; blue oval circles show bacterial cells from the *Bacteroides* genus; yellow, purple and green oval circles indicate other bacteria; DPP4: dipeptidyl peptidase 4; h: host; m: microbial; GLP-1: glucagon-like peptide-1; GIP: glucose-dependent insulinotropic polypeptide; arrows indicate activations. Bars represent inhibitory interactions

Wang and colleagues demonstrate a specific mechanism that may explain the lowered efficacy of antidiabetic DPP4 inhibitors.^1^ The authors found that specific bacteria in the gut microbiome produce enzymes that, upon inflammation or metabolic disorders, can transit through the intestinal wall and operate similarly to their host counterparts (isozymes). Such a microbial enzyme identified was mDPP4. In the host, the hDPP4 isozyme normally degrades GLP-1 and glucose-dependent insulinotropic polypeptide (GIP) hormones, collectively called endogenous incretins as they stimulate insulin secretion in the presence of glucose (Fig. 1). These peptides are secreted from intestinal cells in response to nutrition, and can also inhibit glucagon release from the pancreas, thereby further lowering blood glucose levels (Fig. 1). hDPP4 activity hence stabilizes blood glucose levels by suspending the action of incretins. Using bioinformatic and chemoproteomic analyses, another recent study has also identified a functional microbial homolog of hDPP4.^4^ Revealing the shared function of the host and microbial DPP4 isozymes (hDPP4 and mDPP4) raised the question whether current therapeutic treatment against hDPP4 (e.g., using sitagliptin) also works against mDPP4. Activity assays and structural studies performed by Wang and colleagues showed that the microbial isozyme cannot be inhibited with sitagliptin, necessitating a fundamentally novel therapeutic approach, in which both DPP4 isozymes (hDPP4 and mDPP4) are simultaneously inhibited to get a higher efficacy.^1^ Indeed, co-administration of a newly developed, specific mDPP4 inhibitor, Dau-d4 (a daurisoline derivative), and sitagliptin could further decrease blood glucose levels.

Understanding how the gut microbiota influences host physiology remains a fundamental problem in biology with significant medical implications.^5^ It has been known for long that certain gut microbiota members (often belonging to the *Bacteroides* genus) possess enzymes that have the same or similar functions to human enzymes even with substantially different amino acid sequences. Identifying such isozymes is highly challenging. Using a newly established enzyme activity screening platform, Wang and colleagues tested stool-derived ex vivo microbial communities and identified more than 70 bacterial enzymes with functional activity.^1^ Among these microbial enzymes, mDPP4 was the top hit statistically. The detailed analysis of mDPP4 pointed out that it is mostly produced by *Bacteroides spp* (predominantly by *Bacteroides thetaiotaomicron* strains). A medically relevant observation was that these mDPP4 proteins normally do not cross the gut barrier. However, upon local inflammation or metabolic disorders (conditions associated with T2D), there is a patchy breakdown of the epithelial barrier causing the so-called leaky gut phenomenon when luminal content, including mDPP4, enters the gut mucosal layer and degrades GLP-1. This condition-specific effect of mDPP4 is useful for targeting it, as it means under normal conditions mDPP4 is in the gut lumen and probably has no physiological role. But upon a leaky gut situation, mDPP4 enters the host and its pharmacological inhibition could increase the response rate for sitagliptin in T2D patients.

One of the major strengths of the study was to demonstrate that a group termed sitagliptin low responders (SLRs) has a considerably higher fecal DPP4 activity. However, it remains unanswered whether plasma GLP-1 activity is also changed in these SLR patients. In addition, measuring fecal DPP4 activity may predict the therapeutic potential of sitagliptin treatment in T2D patients. Another clinical relevance of this observation is the importance of a healthy gut during sitagliptin treatment as mDPP4 could not cause problems in a normal epithelial barrier. Hence, diet and probiotic supplements that support the healthy epithelial layer could increase the efficiency of sitagliptin treatment. Thus, probably clinical intervention studies will be based on the detailed mechanisms uncovered by Wang and colleagues. Together, these results uncover a general mechanism that underlies the variable response in certain patients to an antidiabetic medication,^1,4^ and emphasize the importance of developing therapies that simultaneously target a gut microbial and host isozyme pair to achieve greater clinical effectiveness.^1^

Appreciating the role the microbiome plays in normal physiology and pathology^1,4^ has at least three important implications. First, factors that seriously change the composition of the gut microbiome can heavily influence host homeostasis and cause disease. For example, an antibiotic treatment or unbalanced nourishment can lead to the overgrowth of a certain bacterial taxon in the gut microbiome, and the enzyme produced by this community enhances or weakens the function of its host counterpart to pathological levels. Second, circumstances causing damage to the gut structure (e.g., exposure to toxins, inflammation, aging or certain infectious diseases) may enable the transit of bacterial metabolites and proteins with undesired biological activity through the intestinal wall. Third, future pharmacological innovations should consider the fact that several protein targets share redundant roles with their corresponding microbial counterparts even if the latter display no significant homology at the level of amino acid sequence. In this case, inhibitors for both host and bacterial proteins should be simultaneously developed, to reach sufficient efficacy. Alternatively, further studies should focus on developing inhibitors with dual activity against both hDPP4 and mDPP4. Such a combined treatment, supported by establishing and keeping a healthy gut microbiota and gut epithelial barrier, is critical to restore and maintain normal physiology in the host.
